# Milky Ascites: A Diagnostic Dilemma

**DOI:** 10.7759/cureus.34008

**Published:** 2023-01-20

**Authors:** Baha Aldeen Bani Fawwaz, Arooj Mian, Gurdeep Singh, Aimen Farooq, Abu Hurairah

**Affiliations:** 1 Internal Medicine, AdventHealth Orlando, Orlando, USA; 2 Gastroenterology and Hepatology, AdventHealth Apopka, Apopka, USA

**Keywords:** cirrhosis, lymphatic abnormalities, peritoneal fluid, ascites, abdominal paracentesis, chyloperitoneum, milky ascites, chylous ascites

## Abstract

Chylous ascites is a milky-appearing, triglyceride-rich fluid within the abdominal cavity. It is a rare finding that arises from the disruption of the lymphatic system and can be caused by a wide variety of pathologies. Here, we present a diagnostically challenging case of chylous ascites. In this article, we discuss the pathophysiology and various etiologies of chylous ascites, explore the diagnostic tools available, and highlight the management strategies implemented in this rare finding.

## Introduction

Lymphatic fluid is formed mainly from the excess non-absorbed portion of the body’s extravascular fluid, and it comprises substances such as proteins, minerals, and triglycerides. All of the body’s lymph is drained through a network of vessels called the lymphatic system before being returned to the blood vasculature [1]. Defective drainage of the lymph can lead to its accumulation in any of the body’s three serous cavities, i.e., the peritoneum as chyloperitoneum, the thoracic cavity as chylothorax, or the pericardium as chylopericardium. Chyloperitoneum, also known as chylous ascites, is defined as the presence of turbid, milky-appearing, triglyceride-rich fluid in the abdominal cavity [2]. Although the accurate incidence of this pathology remains undisclosed, it is considered rare [2,3]. The pathophysiology of chylous ascites relates primarily to the disruption of lymphatic drainage. Etiologically, this can be due to congenital anomalies, trauma, or secondary to local or systemic diseases [3,4]. Here, we describe a challenging case of chylous ascites and provide a suggestive approach to this finding.

## Case presentation

**Figure 1:**
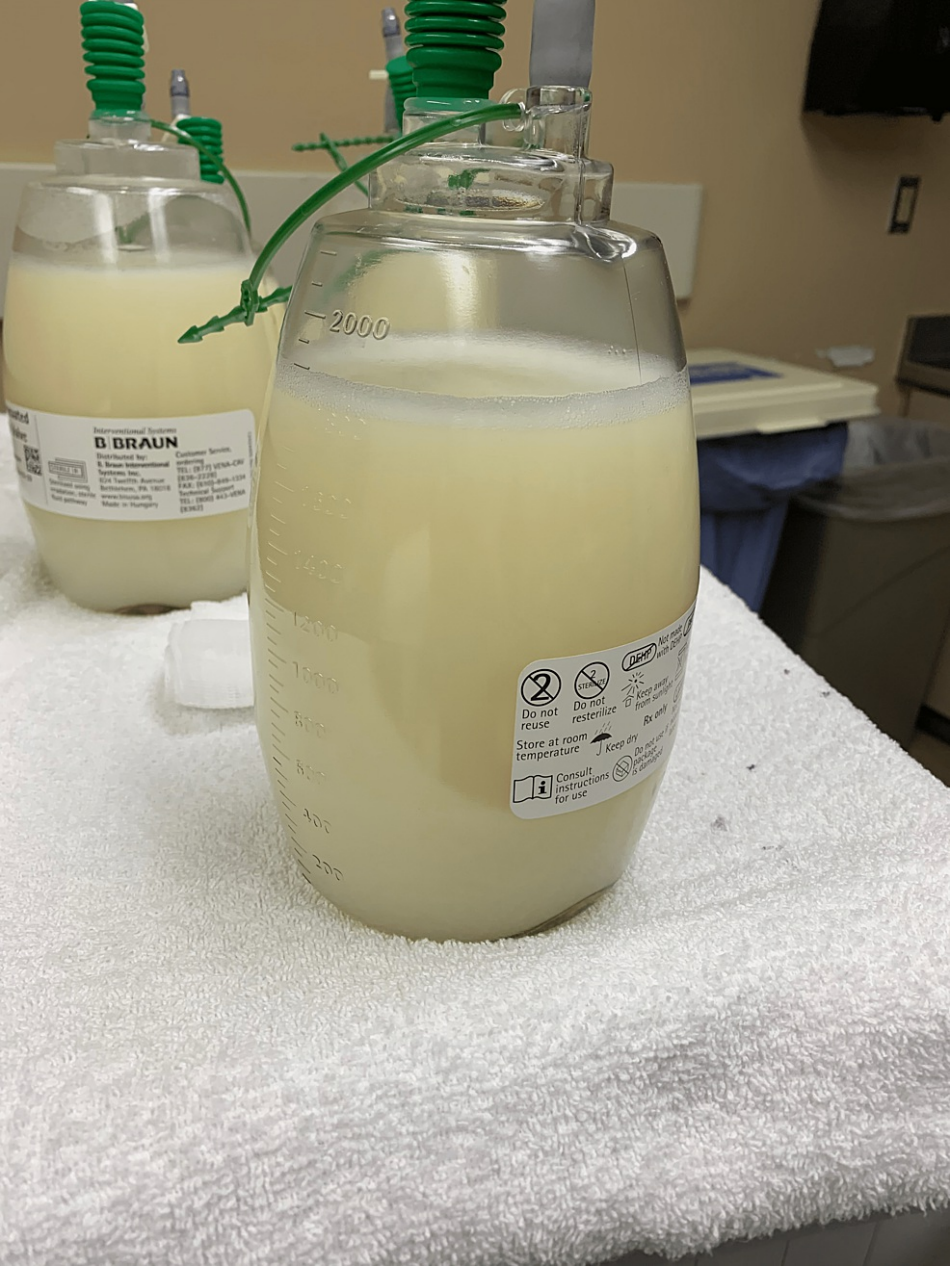
Paracentesis revealed milky and turbid-appearing ascitic fluid

A 35-year-old male with a past medical history of non-Hodgkin's lymphoma in remission, acquired immunodeficiency syndrome (AIDS) on highly active antiretroviral therapy (HAART), immune reconstitution inflammatory syndrome (IRIS), and disseminated Mycobacterium avium complex (MAC) infection was transferred to our tertiary care center for evaluation of large recurrent chylous ascites of unclear etiology. The patient initially presented six months ago with abdominal distension and was found to have ascites. He had no history of hepatitis, jaundice, signs of liver dysfunction, recent trauma, or surgery. He had been undergoing paracentesis every two weeks while being evaluated for an underlying etiology. Workup to evaluate for chronic liver disease and cirrhosis had been unremarkable and a recent liver biopsy showed no evidence of hepatic fibrosis. The ascitic fluid gradually became milky and more turbid four weeks prior to presentation, as depicted in Figure 1. He underwent a paracentesis two days prior to the transfer, which revealed ascitic fluid with an elevated triglyceride level of 232 mg/dL, as depicted in Table 1.

**Table 1:** Ascitic fluid analysis MTB: Mycobacterium tuberculosis; PCR: polymerase chain reaction.

Test	Result
Color	White
Clarity	Cloudy
Nucleated cells	235
RBCs	<2000
Neutrophils	4
Lymphocytes	8
Monocytes	10
Macrophages	76
Mesothelial	2
Eosinophils	0
Albumin	<0.2
Total protein	0.6
Triglycerides	331
Lactate dehydrogenase	46
MTB complex PCR qualitative	Not detected

Upon arrival at our tertiary care hospital, further workup was initiated. Initial laboratory investigations revealed a total protein level of 3.9 g/dL, serum albumin of 2.5 g/dL, serum globulin of 1.4 g/dL, serum calcium of 4.3 mg/dL, corrected calcium of 5.5 mg/dL, alanine aminotransferase (ALT) of 73 units/L, aspartate aminotransferase (AST) of 36 units/L, a CD4 count of 94, and an undetectable HIV viral load. The hepatitis panel was negative and the urinalysis showed trace albuminuria.

A magnetic resonance imaging (MRI) of the abdomen and pelvis with and without intravenous (IV) contrast revealed large-volume ascites with a normal liver architecture and patent hepatic vasculature. There was no evidence of cirrhosis, lymphadenopathy, intra-abdominal malignancy, and lymphoproliferative disorders. An echocardiogram revealed an ejection fraction of 65-70% without evidence of right ventricular dysfunction. A repeat paracentesis revealed white and cloudy ascitic fluid with 235 nucleated cells (4% neutrophils, 8% lymphocytes, 10% monocytes, 76% macrophages, and 2% mesothelial cells), indicating the absence of spontaneous or secondary bacterial peritonitis, a serum ascites albumin gradient (SAAG) of 2.2 consistent with portal hypertension, an albumin level <0.2 g/dL, a total protein level of 0.6 g/dL, and a triglyceride level of 331 mg/dL, reconfirming chylous ascites. Furthermore, bacterial culture with gram stain, acid-fast bacillus (AFB) smear, Mycobacterium complex PCR, and flow cytometry of the ascitic fluid returned negative. Lastly, ascitic fluid histopathology was negative for malignant cells.

Subsequently, a nuclear medicine lymphoscintigraphy scan was performed to identify any blockage of lymphatic flow. The lymphoscintigraphy demonstrated normal lymphatic flow to the level of the pelvis. However, there was no lymphatic flow beyond the pelvic lymph nodal station, out to 2.75 hours post-injection, including the liver, which showed no activity. These findings suggested a possible lymphatic obstruction or lymphatic leakage somewhere in the pelvic region. Strangely, however, no obvious lymphadenopathy was noted on the CT and MRI imaging in this particular region of concern. The role of pelvic lymph node biopsies to evaluate for a possible lymphoma was considered. However, this was not feasible as no lymph node enlargement was seen on imaging.

Given the circumstances, we opted to manage our patient conservatively. He was treated with dietary modifications including a high-protein and low-fat diet with medium chain triglyceride (MCT) oil. Additionally, he received diuretic therapy and empiric MAC treatment and was continued on his HAART therapy. His symptoms gradually improved and he was discharged in stable condition. The patient was advised to follow up in the outpatient setting where he will continue to undergo paracentesis as needed.

## Discussion

The approach to managing chylous ascites begins with confirming the diagnosis. Chylous ascites is defined as ascitic fluid with a triglyceride level greater than 200 mg/dL [5]. The causes of chylous ascites can be broadly classified into traumatic, such as post-operative chyloperitoneum [6], and atraumatic etiologies. Atraumatic causes are numerous and include congenital abnormalities, infections, malignancies, and inflammatory disorders. As congenital etiologies are predominantly diagnosed in pediatric populations, it was not a part of our differential diagnosis. Since our patient was immunocompromised with a history of malignancy, he underwent an infectious and malignancy workup. Unexpectedly, these diagnostic studies were unremarkable. Furthermore, a recent evaluation for chronic liver disease had been unremarkable and the liver biopsy revealed no evidence of cirrhosis. Moreover, an MRI of the abdomen and pelvis revealed a normal liver architecture and patent hepatic vasculature. At this point, investigating rare causes of chylous ascites became a valid approach. The lymphoscintigraphy suggested a possible lymphatic obstruction or leakage in the pelvic region but without evidence of lymph node enlargement on imaging. At last, no obvious etiology was found that could explain the patient’s chylous ascites.

Management of chylous ascites should be targeted against the possible etiology. While workup is initiated, supportive treatment with as-needed paracentesis and diet optimization should be pursued simultaneously. The goal of nutrition therapy is to maintain nutritional status, replace fluid losses, and decrease the net production of chyle. Although there is no evidence supporting the superiority of one nutritional therapy over another, a high-protein and low-fat diet with MCT is recommended. This diet prevents the conversion of long-chain triglycerides (LCT) into their substitutes, which are then transported through the lymphatic ducts. It is worth mentioning that the use of MCT oil is not recommended in cirrhotic patients because it can lead to coma [7].

Our decision to empirically treat for a MAC infection was supported by the immunocompromised status of our patient, the absence of other apparent etiologies, and the possibility of false negative test results.

We will continue to investigate possible etiologies leading to our patient’s chylous ascites including an evolving malignancy and medication-related effects until a clear diagnosis is reached. Until then, this diagnostically challenging case remains a mystery.

## Conclusions

Chylous ascites is a rare clinical manifestation and can be seen with pathologies that disrupt the lymphatic drainage of the peritoneum. A comprehensive evaluation should be performed for all patients presenting with chylous ascites, including investigations for infections, liver diseases, and malignancy. Management involves treating the underlying etiology, if identified, and supportive therapy with diuresis, as-needed paracentesis, and dietary modification. Despite these efforts, the overall prognosis of patients with chylous ascites remains poor.
